# Mild Cognitive Impairment Detection System Based on Unstructured Spontaneous Speech: Longitudinal Dual-Modal Framework

**DOI:** 10.2196/80883

**Published:** 2026-01-15

**Authors:** Yu-Shan Liao, Thiri Wai, Ting-Yun Liao, Ho-Ling Chang, Yu-Ling Chang, Li-Chen Fu

**Affiliations:** 1 Graduate Institute of Networking and Multimedia, National Taiwan University, Taipei, Taiwan; 2 Department of Computer Science and Information Engineering, National Taiwan University, No. 1, Sec 4, Roosevelt Rd, Taipei, Taiwan, 886 0233663366; 3 Department of Psychology, National Taiwan University, Taipei, Taiwan; 4 Center of Artificial intelligence Research, University of Tsukuba, Tsukuba, Ibaraki, Japan

**Keywords:** mild cognitive impairment, longitudinal speech analysis, multimodal fusion, Alzheimer disease detection, deep learning, autobiographical memory test

## Abstract

**Background:**

In recent years, the incidence of cognitive diseases has also risen with the significant increase in population aging. Among these diseases, Alzheimer disease constitutes a substantial proportion, placing a high-cost burden on health care systems. To give early treatment and slow the progression of patient deterioration, it is crucial to diagnose mild cognitive impairment (MCI), a transitional stage.

**Objective:**

In this study, we use autobiographical memory (AM) test speech data to establish a dual-modal longitudinal cognitive detection system for MCI. The AM test is a psychological assessment method that evaluates the cognitive status of subjects as they freely narrate important life experiences.

**Methods:**

Identifying hidden disease-related information in unstructured, spontaneous speech is more difficult than in structured speech. To improve this process, we use both speech and text data, which provide more clues about a person’s cognitive state. In addition, to track how cognition changes over time in spontaneous speech, we introduce an aging trajectory module. This module uses local and global alignment loss functions to better learn time-related features by aligning cognitive changes across different time points.

**Results:**

In our experiments on the Chinese dataset, the longitudinal model incorporating the aging trajectory module achieved area under the receiver operating characteristic curve of 0.85 and 0.89 on 2 datasets, respectively, showing significant improvement over cross-sectional, single time point models. We also conducted ablation studies to verify the necessity of the proposed aging trajectory module. To confirm that the model not only applies to AM test data, we used part of the model to evaluate the performance on the ADReSSo dataset, a single time point semistructured data for validation, with results showing an accuracy exceeding 0.88.

**Conclusions:**

This study presents a noninvasive and scalable approach for early MCI detection by leveraging AM speech data across multiple time points. Through dual-modal analysis and the introduction of an aging trajectory module, our system effectively captures cognitive decline trends over time. Experimental results demonstrate the method’s robustness and generalizability, highlighting its potential for real-world, long-term cognitive monitoring.

## Introduction

Alzheimer disease (AD) is a neurodegenerative disorder often associated with memory decline, language impairment, and a diminishing capacity to perform daily activities independently. However, AD remains incurable. Current research indicates that approximately 47 million people were diagnosed with dementia in 2015 [1], and it is expected that the number will triple by 2050 [2]. Such forecasts pose a significant burden on both patients’ families and health care systems.

Within the spectrum of cognitive health, an intermediary stage exists between a state of normalcy and the onset of AD, denoted as mild cognitive impairment (MCI). Patients with MCI do not exhibit significant cognitive symptoms compared with those with AD. Those patients are still capable of performing daily activities independently. According to the study by Petersen et al [3], an estimated 16% of individuals with MCI progress to dementia within a year. Therefore, early identification of MCI holds significant value in instigating interventions aimed at impeding the advancement of dementia. However, the detection of MCI presents a notable challenge due to the subtle manifestation of its symptoms.

While biomarkers have demonstrated sensitivity in identifying MCI, such as positron emission tomography and magnetic resonance imaging [4,5], many of them necessitate invasive procedures. The associated costs of data collection for such methods are relatively high. Conversely, leveraging voice data for cognitive assessment offers a simpler and more suitable approach for long-term monitoring of cognitive status. Our approach relies on using autobiographical memory (AM) test data as the primary input [6], wherein interviewers engage participants in a series of questions and answers regarding their life experiences. This method, resembling a casual conversation, is more approachable than other psychological test methods because it reduces the testing stress experienced by participants.

Longitudinal analysis for MCI detection based on speech data remains relatively unexplored compared with other data types, such as magnetic resonance imaging, resulting in a scarcity of research in this area. The research proposed by Al-Hameed et al [7] focuses on extracting solely acoustic features from speech data collected across multiple visits from DementiaBank, which primarily pertains to speech associated with picture description tasks. They aim to predict participants’ Mini-Mental State Examination scores and cognitive labels. Following feature selection, they used support vector machine or stochastic gradient descent for prediction. Integrating data from 3 visits achieved a 90% accuracy in distinguishing between healthy control (HC) and MCI in a binary classification task. However, due to label imbalance in the dataset, accuracy as a metric may not be entirely equitable. This study demonstrates that incorporating features from previous visits enhances prediction accuracy for subsequent visits.

Using the same dataset, another survey by Ammar et al [8] concentrates on extracting a wide range of linguistic features related to fluency and richness for Mini-Mental State Examination score prediction. However, unlike the former study, they do not leverage the correlation between previous visits. In the research proposed by Laguarta and Subirana [9], 16 physiological biomarkers were identified from speech data, represented in a personalized subject feature map. This feature map is capable of illustrating and monitoring cognitive changes over time. Achieving 93.6% accuracy in the ADReSS datasets, this study is the only one among them that visually depicts cognitive decline.

Yamada et al [10] used phone calls for long-term participant tracking, manually transcribing the conversations to extract linguistic features. They focused on extracting topic and word repetition features across different calls. Their model achieved an area under the receiver operating characteristic curve (AUROC) of 0.91 in classifying between AD and HC. In our previous work proposed by Chang et al [11], the author first extracted traditional acoustic feature sets, extended Geneva Minimalistic Acoustic Parameter Set [12], from AM audio files collected across multiple visits. The author then used TabNet [13] to learn the cognitive information embedded within these feature sets. To further enhance the model, an aging alignment loss was applied, encouraging cognitive representations in the latent space to be closer to each other. The approach achieved an accuracy of 0.66 and an *F*_1_-score of 0.64.

In this work, we use AM data as the primary input for our model. We analyze the AM data from both linguistic and acoustic perspectives and further establish an aging trajectory module (ATM). By introducing the ATM, which captures cognitive decline across different time points through direction embeddings, our framework provides more representative modeling of longitudinal changes. The key contributions of this work are summarized as follows:

1. Unlike most studies focusing on single time point analysis, this work introduces a longitudinal framework using AM speech data to track cognitive changes over time.
2. The system jointly analyzes linguistic and acoustic features from unstructured AM speech, offering a richer representation of cognitive status than unimodal approaches.
3. Proposed ATM, which models cognitive decline by aligning temporal features across visits through a specially designed alignment loss applied to direction embeddings.

## Methods

### Problem Setting

There are 2 problem settings in this work: longitudinal type and cross-sectional type. The longitudinal problem setting is our main focus. The cross-sectional problem setting evaluates whether our model is general enough to tackle different tasks of detecting cognitive impairment disease detection tasks.

#### Longitudinal Problem Setting

In our problem setting, we have the dataset $D=\left\{D_{1},D_{2},\ldots ,D_{V}\right\}$with the corresponding label set $L=\left\{L_{1},L_{2},\ldots ,L_{V}\right\}$, where *V* means the total visit times, and *D*_*k*_ means the dataset at the *k*th visit. Each *D*_*k*_ is expressed as $D_{k}=\left\{x_{1,k},\;x_{2,k},\;\ldots ,\;x_{N,k}\right\}$, where *N* represents the number of the subjects involved in the test, and$x_{i,k}=\left\{x_{i,k}^{1},\;x_{i,k}^{2},\;\ldots ,x_{i,k}^{M}\right\}$ represents the *M* stories provided by subject *i* at the *k*th visit; note that the number of *M* may vary across different visits. The corresponding labels set $L_{k}=\left\{l_{1,k},\;l_{2,k},\;\ldots ,\;l_{N,k}\right\}$ shows the cognitive status of each subject, with each *l_i,k_* indicating whether the subject is an HC or has MCI.

During the data preprocessing, we extract the feature set $\left\{x_{i,k}^{m,a},\;x_{i,k}^{m,t},\;x_{i,k}^{m,l}\right\}$ from the raw audio $x_{i,k}^{m}$, where $x_{i,k}^{m,a},\;x_{i,k}^{m,t},\;x_{i,k}^{m,l}$ represents the acoustic feature set, textual data, and linguistic feature set, respectively. Subsequently, the model input is the sequential visit data defined in equation 1, where $x_{i,1}^{m_{1}}\in \;x_{i,1}$, $x_{i,2}^{m_{2}}\in \;x_{i,2}$, and$\;\;x_{i,V}^{m_{V}}\in \;x_{i,V}$.


(1) $$x_{i,1}^{m_{1}},\;x_{i,2}^{m_{2}},\ldots ,\;x_{i,V}^{m_{V}}=\left\{\begin{array}{c} \left\{x_{i,1}^{m_{1},a},\;x_{i,1}^{m_{1},t},\;x_{i,1}^{m_{1},l}\right\},\;\\ \left\{x_{i,2}^{m_{2},a},\;x_{i,2}^{m_{2},t},\;x_{i,2}^{m_{2},l}\right\},\\ \ldots ,\;\\ \left\{x_{i,V}^{m_{V},a},\;x_{i,V}^{m_{V},t},\;x_{i,V}^{m_{V},l}\right\} \end{array}\right\}$$

It is worth noting that stories from each visit are randomly paired across visits within each subject. This design ensures that the model learns subject-level temporal progression rather than relying on similarities driven by specific story topics or linguistic content. Random pairing is not an arbitrary source of noise but a deliberate mechanism aligned with our longitudinal modeling objective. Because story content varies substantially across visits and participants, enforcing semantic matching or averaging all embeddings within a visit would bias the model toward topic-dependent features, flatten meaningful variability in linguistic complexity, and increase the risk of overfitting to specific story topics. In contrast, random pairing operates strictly within each subject, preserving the temporal order while exposing the model to multiple sampled transitions across training epochs. This provides a richer distribution of temporal transitions, enabling the framework to learn visit-level cognitive progression directions that remain robust to story content and reflect each subject’s distribution of linguistic changes over time.

Formally, our model can be viewed as a mapping function $f^{W}$, where *W* represents the set of learnable parameters. The function $f^{W}$projects the input sequence $\left\{x_{i,1}^{m1},x_{i,2}^{m2},\ldots ,x_{i,v}^{mv}\right\}$ into the label of the last visit $l_{\left(i,V\right)}$. In this study, the maximum number of visits per subject, *V*, is 3, following the available structure of the dataset.

#### Cross-Sectional Problem Setting

In the cross-sectional problem setting, the primary difference from the longitudinal problem setting is that data collected from the same subject at different interview visits are treated as data from independent subjects. Thus, we have the dataset $D=\left\{x_{1},x_{2},\;\ldots ,\;x_{CN}\right\}$ with the corresponding label set $L=\left\{l_{1},l_{2},\;\ldots ,\;l_{CN}\right\}$, where CN is the sum of all subjects from all visits, and *x_i_* represents the raw audio for the subject *i* with the cognitive label *l_i_*. The cognitive label *l_i_* indicates that the subject is either an HC or under the targeted condition (MCI or AD). During the data preprocessing, we extract the feature set $\left\{x_{i}^{a},x_{i}^{t},x_{i}^{l}\right\}$ from the raw audio *x_i_*. Our cross-sectional model aims to map the model input $\left\{x_{i}^{a},x_{i}^{t},x_{i}^{l}\right\}$ into the cognitive label *l*_*i*_.

### Overview

**Figure 1.**
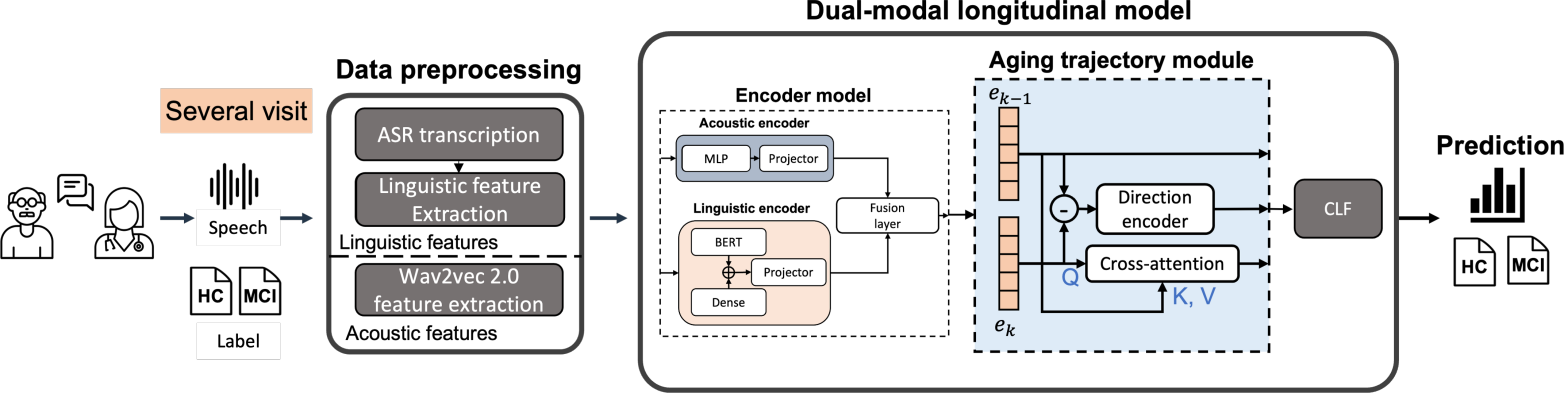
Overall framework of the proposed longitudinal multimodal model. The model consists of two main modules: (1) encoder model, combining a CNN-based acoustic encoder and a BERT-based linguistic encoder to generate fused multimodal embeddings; and (2) aging trajectory module, which models longitudinal changes between consecutive visits using a direction encoder and cross-attention before classification. ASR: automatic speech recognition; BERT: bidirectional encoder representation for transformer; CLF: classifier; CNN: convolutional neural network; HC: healthy control; MCI: mild cognitive impairment.

The system overview is shown in Figure 1. The goal of the system is to detect the cognitive condition of subjects, specifically whether they have MCI, using speech data. Before training, the speech data first undergo data preprocessing, which involves converting the audio files to text using automatic speech recognition (ASR), extracting additional linguistic features, and deep learning–based acoustic feature extraction. These steps generate additional text data and acoustic data, which are then intertwined to form dual-modal data pairs serving as the input for the model training. In addition, to effectively capture the features of aging and enhance the detection of potential MCI, the model input should have speech data for this time as well as points of follow-up data. This philosophy leads to our proposed longitudinal analysis for MCI detection.

### Data Preprocessing

Before the data are input into the model, they need to be preprocessed to ensure data quality and to extract useful features. After data collection is completed, given that the data are based on the entire interview process of each subject during a single visit, we first segment each story recording from the interview. To prepare the audio file of each subject, we segment the audio files based on the story topics provided by the subject, cutting the corresponding recall and probing audio files.

### Acoustic Feature Extraction

**Figure 2.**
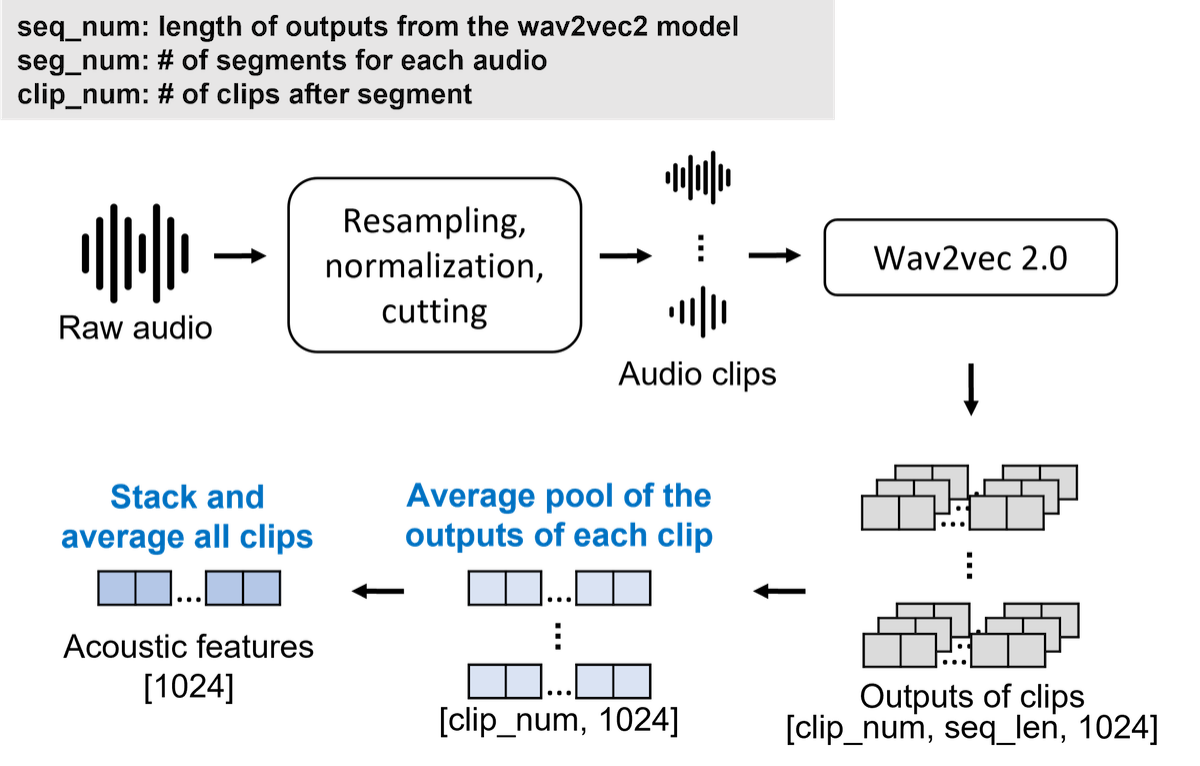
Overview of acoustic preprocessing. Each raw audio recording is divided into multiple fixed-length clips and processed by the wav2vec2.0 feature extractor to obtain frame-level representations. For each segment, the outputs from all clips are stacked and averaged to produce a single clip-level feature. The averaged clip features are then pooled across segments to generate the final visit-level wav2vec2.0 embedding, which is used as input to the acoustic encoder.

The preprocessing of audio data is shown in Figure 2. First, the notation $x_{i,k}^{m}$ denotes a raw audio waveform, which means the *m*th audio file of the subject *i* collected at the *k*th interview visit undergoes basic processing, including normalization and resampling. Normalization is done using zero-mean unit-variance normalization. Next, the audio is segmented into *t*-second clips with a 5-second overlap. Each audio file can be denoted as $x_{i,k}^{m}=\{x_{i,k}^{m,1},x_{i,k}^{m,2},\ldots ,x_{i,k}^{m,seg\_num}\}$, where seg_num represents the number of clips to which the raw audio file can be divided, and the total number of segments varies for each audio file.

In our research, wav2vec 2.0 model [14] is used as an acoustic feature extractor, and we leverage the pretrained model available on Hugging Face. Using the wav2vec 2.0 model in advance to extract deep acoustic representation features offers the advantage of saving memory space and making the subsequent model structure lighter, thereby increasing training efficiency. Each clip is fed into the wav2vec 2.0 model, producing an output with dimensions [seq_len, 1024], where seq_len is determined by the convolution layer in wav2vec 2.0 and the clip length, which is computed by dividing the clip length and the multiplication of the stride length in the convolution layers. After obtaining the output of each clip, the first average pooling is performed, resulting in a 1024-dimensional acoustic feature for each clip. Then, all clip outputs are stacked together, and a final average pooling is conducted to obtain the final acoustic features $x_{i,k}^{m,a}$ of the entire raw audio file; hence, the dimension of $x_{i,k}^{m,a}$ is 1024.

### Linguistic Feature Extraction

**Figure 3.**
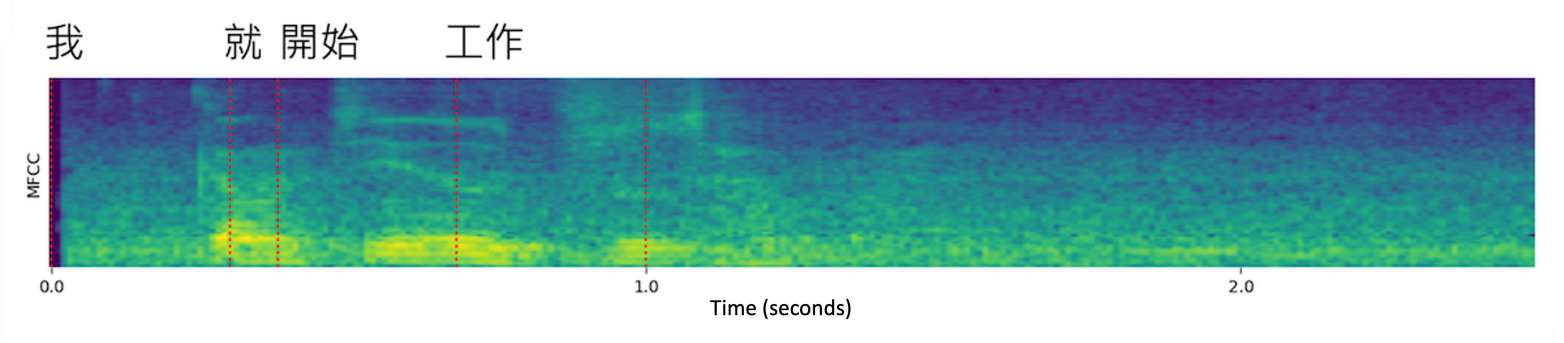
Example of how the result of whisper-timestamped, where the timestamp of the Mandarin speech is aligned with the transcript, is used for the temporal segmentation to extract linguistic features. MFCC: mel frequency cepstral coefficients.

The preprocessing of linguistic data can be divided into 2 stages: generating the transcript and extracting features from the generated text. Since not all audio files in the dataset have manual transcripts, we use ASR to generate the transcripts for our research. We use the open-source tool kit “whisper-timestamped,” which provides both timestamps and the corresponding text, as illustrated in Figure 3. Previous research [15-17] has confirmed that pauses are an indicator in AD-related diseases, where patients with MCI or AD tend to exhibit more frequent and longer pauses. In our study, we classify pauses based on the interval duration between sentences into 3 types: short pause (duration <0.5 seconds), medium pause (duration between 0.5 and 2 seconds), and long pause (duration >2 seconds) [18]. In addition to pauses, we calculate the mean length of utterance, which is defined as the average number of words spoken in a sentence. In this step, we will generate transcripts with pause information and 4 numerical linguistic features. Besides pause information, we provide additional syntax information to enrich the linguistic features. Previous research [19] has shown that the sentence structures used by individuals with AD and HC are different. Therefore, we use part-of-speech (POS) tagging in this study. After generating the text, we use the open-source tool kit “ckipnlp” for word parsing and POS tagging. The text segments are then categorized into 5 POS types: noun,

verb, adjective, pronoun, and other. In this step, we count the total number of these POS taggers.

In addition, due to the nature of the AM test, subjects can share stories on unrestricted topics. To facilitate the model to capture more meaningful information from the transcripts, we apply the topic insertion technique proposed by our previous work [11,18]. This technique is to add a sentence with the mentioned topic at the beginning of the transcript, such as “I’m going to share a story of...” The tester specifies the topic during data collection. This step helps ensure that the subsequent transcript is thematic. After the whole preprocessing of linguistic data, from the raw audio $x_{i,k}^{m}$, we finally obtain the transcripts with topics and pauses $x_{i,k}^{m,t}$, along with 9 additional linguistic features $x_{i,k}^{m,l}$, which include mean length of utterance; the number of short, medium, and long pauses; and 5 types of POS tags.

### Encoder Module

#### Acoustic Encoder

During the data preprocessing stage, 1024-dimensional deep learning–based acoustic features, denoted as $x_{i,k}^{m,a}$, were extracted using the wav2vec 2.0 model. These high-level representations were then passed to a lightweight convolutional neural network–based acoustic encoder designed to capture hierarchical temporal-spectral patterns. The encoder consists of three 1D convolutional layers with kernel size 3 and stride 1, each followed by batch normalization, rectified linear unit activation, and max-pooling with a kernel size and stride of 2, progressively reducing the feature resolution by a factor of 8. The convolutional layers use 16, 32, and 64 filters, respectively, to model increasingly abstract local dependencies in the acoustic embedding. The resulting feature maps are flattened and projected through a fully connected layer that maps the representation to a 128-dimensional latent space. This projection enhances computational efficiency, mitigates overfitting, and provides a compact and informative acoustic embedding $a_{i,k}^{m}$ for multimodal fusion.

#### Linguistic Encoder

In the linguistic encoder, the ASR transcript $x_{i,k}^{m,t}$ and 9-dimension features $x_{i,k}^{m,l}$ generated during data preprocessing are sent to the BERT [20] model and a linear layer, respectively, for initial encoding. During data preprocessing, to ensure that the subjects’ speech has a thematic structure, we add a topic at the beginning of the transcript. In BERT, we borrow the concept of segment embeddings from the original paper [20]. Here, we refer to it as “context embedding” to enhance the model’s understanding of the topic and content. We divide the transcript into 2 parts: topic and context. The topic part is labeled as 0, and the context part as 1. These values are then encoded into context embeddings *E*_*t*_ and *E*_c_ through a learning embedding layer. This design helps the model determine whether there is a sufficient correlation between the topic and content. Research in AM tests indicates that episodic memory significantly declines in patients with cognitive impairments [21,22]. Therefore, we can assess the subject’s cognitive state by examining whether the subject’s story contains details related to the topic.

**Figure 4.**
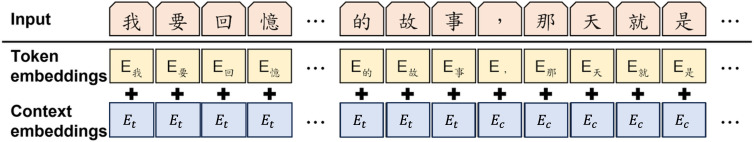
Overview of the BERT embedding. Each input token is first mapped to a token embedding, which is combined with additional context embeddings to capture both semantic meaning and conversational context. BERT: bidirectional encoder representation for transformer.

As illustrated in Figure 4, context embeddings are element-wise added to the original BERT token embeddings for further processing. A linear layer is used as a numerical encoder for linguistic features $x_{i,k}^{m,l}$. Finally, the output from BERT and the learned numerical embeddings are concatenated and processed through a projector to obtain the final linguistic embedding $t_{i,k}^{m}$.

#### Fusion Layer

**Figure 5.**
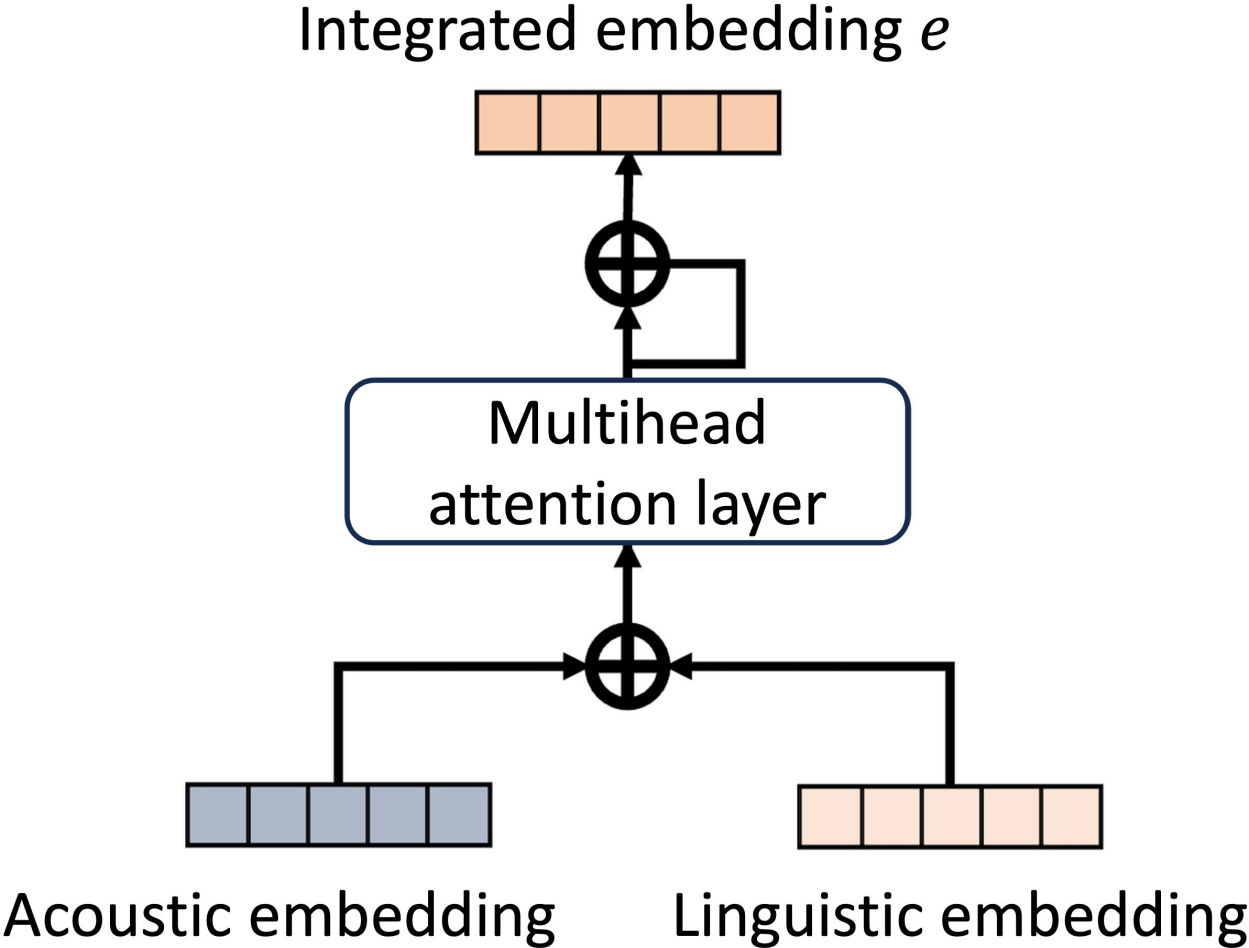
Overview of the fusion layer. Acoustic and linguistic embeddings are integrated through a multihead attention mechanism, producing a joint embedding that captures cross-modal interactions and complementary cognitive cues.

In this study, we adopt a self-attention fusion method to enable the model to capture information between different modalities more effectively. The concept is illustrated in Figure 5. After obtaining the acoustic embedding $a_{i,k}^{m}$ and the linguistic embedding $t_{i,k}^{m}$, these 2 embeddings are added together to form a combined representation, which is then fed into a multihead self-attention layer. Through the calculations of the self-attention layer, we obtain the final integrated embedding $e_{i,k}^{m}$. This design helps the model capture deeper interrelated information between different features.

### Aging Trajectory Module

**Figure 6.**
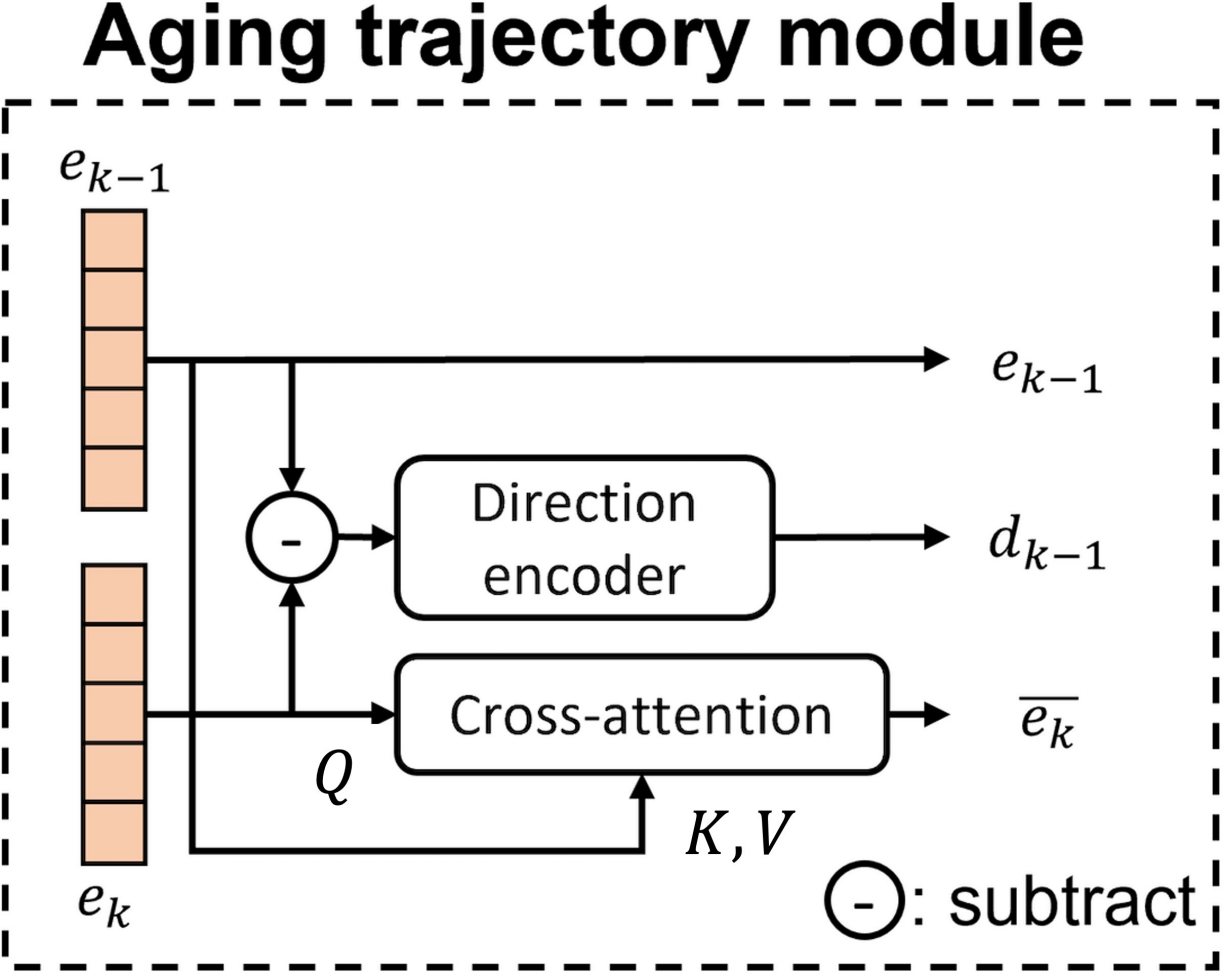
Overview of the aging trajectory module. Consecutive visit embeddings are compared through cross-attention and a direction encoder to capture cognitive differences between visits.

The ATM is a key component of our longitudinal model, designed to capture cognitive changes across multiple visits. The structure is shown in Figure 6. In our study, each subject contributes up to 3 visits. Therefore, the model’s initial input is in the form of a triplet, $\left\{x_{i,1}^{m_{1}},\;x_{i,2}^{m_{2}},x_{i,3}^{m_{3}}\right\}$. After passing them through the encoder model process, this becomes the embedding triplet of the same form, $\left\{e_{i,1}^{m_{1}},\;e_{i,2}^{m_{2}},e_{i,3}^{m_{3}}\right\}$. In this module stage, the embeddings are fed in pairs, such as ${\{e}_{i,1}^{m_{1}},e_{i,2}^{m_{2}}\}$or ${\{e}_{i,2}^{m_{2}},e_{i,3}^{m_{3}}\}$, so that the model focuses on learning the relationship between visits *k* and *k-*1. To model these relationships, we use a cross-attention mechanism, where the embedding from the *k*th visit serves as the query (*Q*), and the embedding from the (*k*-1)th visit serves as the key (*K*) and value (*V*). This mechanism allows us to capture the differences in feature values between 2 visits. The reason for using the *k*th visit embedding as the query and the (*k*-1)th visit embedding as the key and value is that, in practice, we can retrieve information only from the past data and not future data. This approach ensures that the model can access information only from the previous visits, reflecting a realistic scenario where future data are unavailable. By doing so, the model learns to focus on the most relevant past information that can be referenced by the current visit, enabling the tracking of changes and peeking of patterns of cognitive decline over time. This method effectively highlights the differences and similarities between visits, providing a more nuanced understanding of the subject’s cognitive progression.

The concept of contrasts is quite straightforward. We aim to explore a deeper understanding of the relationship between the (*k*-1)th and *k*th visits. To achieve this, we use a cross-attention mechanism. In this setup, the embedding from the *k*th visit is used as the query (*Q*), whereas the embedding from the (*k*-1)th visit serves as the key (*K*) and value (*V*). This mechanism allows us to capture the differences in feature values between 2 visits. The reason for using the *k*th visit embedding as the query and the (*k*-1)th visit embedding as the key and value is that, in practice, we can retrieve information only from the past data and not future data. This approach ensures that the model can access information only from the previous visits, reflecting a realistic scenario where future data are unavailable. By doing so, the model learns to focus on the most relevant past information that can be referenced by the current visit, enabling the tracking of changes and peeking of patterns of cognitive decline over time. This method effectively highlights the differences and similarities between visits, providing a more nuanced understanding of the subject’s cognitive progression.

To quantify the differences between visits, we introduce a direction encoder *f_D_*, composed of several linear layers. The difference between embeddings is projected into a latent space to represent an aging-oriented change vector *d*, formulated as equation 2, where $d_{i,k-1}^{m_{k-1},m_{k}}$ means the direction from ${m_{k-1}}^{th}$ story in the (*k*-1)th visit to the ${m_{k}}^{th}$ story in the *k*th visit. $m_{k}=\left\{1,2,\ldots ,M_{i,k}\right\}$, where $M_{i,k}$ is the total number of stories for subject *i* in the *k*th visit. Similarly, $m_{k-1}=\left\{1,2,\ldots ,M_{i,k-1}\right\}$, where $M_{i,k-1}$ is the total number of stories for subject *i* in the (*k*-1)th visit.


(2) $$d_{i,k-1}^{m_{k-1},m_{k}}=\;f_{D}\left(e_{i,k}^{m_{k}}-e_{i,k-1}^{m_{k-1}}\right)$$

In addition, during the training process, motivated by the research [11,23,24], we introduce 2 types of losses: subject alignment loss and group alignment loss. These alignment losses are designed to align both individual and group targets, helping the direction encoder learn more accurate aging-oriented changes. We expect that by applying these losses, the resulting direction *d* will carry more representative meaning, aiding the longitudinal model in accurately assessing the subject’s cognitive condition.

### Subject Alignment Loss

**Figure 7.**
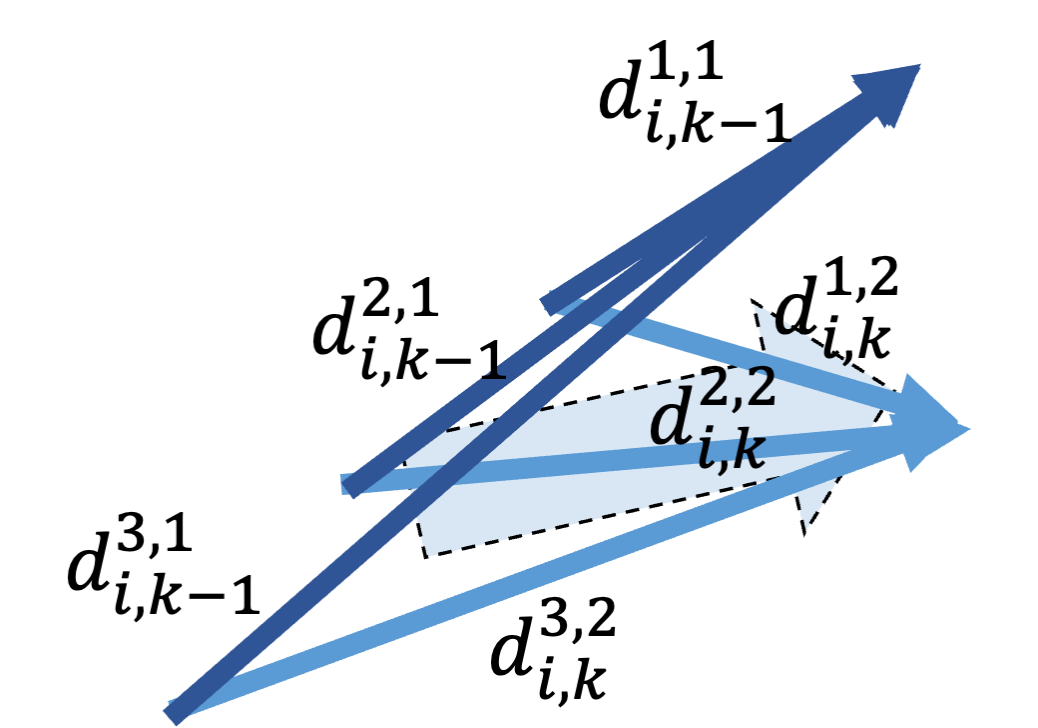
Illustration of the concept for subject alignment loss. It visualizes how the subject alignment loss ensures that the overall trajectory directions of the subject remain consistent, although the internal trajectory of story-level embeddings for the same subject may show slight variations across different visits.

In the subject alignment loss *L_S_*, the concept is to ensure that the same subject’s internal directions are similar. In our problem setting, the stories of the input triplet are randomly paired in different visits. Therefore, it is expected that the directions from the same subject may vary in the latent space. However, the cognitive decline of a subject should be consistent. Figure 7 shows an illustration of the concept.

Therefore, subject alignment loss aims to reduce the differences in direction within the same subject. The loss used here is cosine similarity loss. The process of calculating the subject alignment loss is to first calculate the average subject direction for each visit as shown in equation 2. We then compute the subject alignment loss by summing the alignment loss of all subjects as defined in equation 3. The generalized subject alignment loss is defined as equation 3, where $\overset{-}{d_{i,k-\text{1}}}$ represents the average direction of every audio file in the (*k*-1)th visit to *k*th visit from the subject *i* in the latent space, and *N* represents the total number of subjects. *L_S,i_* represents the subject alignment loss for subject *i*, and $\cos\left(\theta \left(A,B\right)\right)=\frac{A.B}{\left\|A\right\|\left\|B\right\|}$ is the cosine similarity function. In our case, since the dataset contains 3 visits, our *V* is 3, and the loss is applied to the pairs (first visit and second visit) and (second visit and third visit).


(3) $$\overset{-}{d_{i,k-\text{1}}}=\frac{1}{M_{i,k-1}\times M_{i,k}}\sum_{m_{k}=1}^{M_{i,k}}\sum_{m_{k-1}=1}^{M_{i,k-1}}d_{i,k-1}^{m_{k-1},m_{k}}$$

$$\;\;\;\;\;\;\;\;\;\;\;\;\;\;\;\;\;\;\;\;\;\;\;\;\;\;\;\;\;\;\;\;L_{S}=\sum_{i=1}^{N}L_{S,i}$$

(4) $$\;\;\;\;\;\;\;\;\;\;\;\;\;\;\;\;=\;\sum_{i=1}^{N}\sum_{k=2}^{V}\sum_{m_{k}=1}^{M_{i,k}}\sum_{m_{k-1}=1}^{M_{i,k-1}}1-cos\left(\theta \left(\overset{-}{d_{i,k-\text{1}}},d_{i,k-1}^{m_{k-1},m_{k}}\right)\right)$$

### Group Alignment Loss

**Figure 8.**
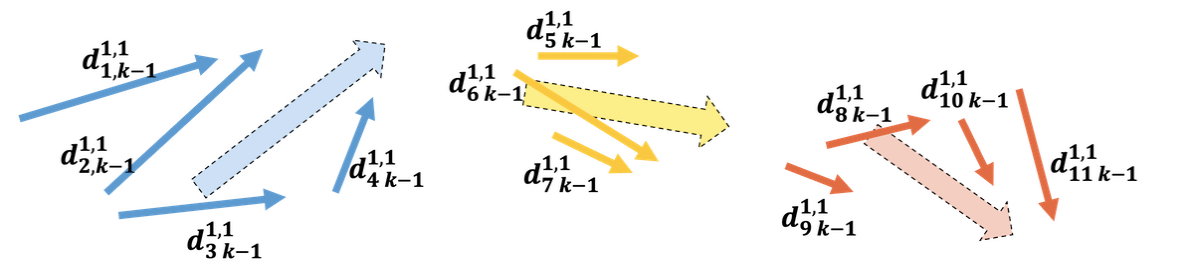
Illustration of the concept for group alignment loss. It visualizes how the group alignment loss enforces consistency in progression directions among subjects sharing the same cognitive label. Blue represents the group from healthy control (HC) to HC, yellow represents the group from HC to mild cognitive impairment (MCI), whereas orange represents the group from MCI to MCI.


In the group alignment loss *L_G_*, the concept is to ensure that subjects with the same cognitive label have similar cognitive progression changes as illustrated in Figure 8. Unlike the subject alignment loss, which focuses on the internal alignment of directions within a subject, the group alignment loss emphasizes the external alignment of directions between different subjects. Specifically, we compare the cognitive change directions of subjects with the same label transitions. Here, we use the Supervised Contrastive Loss (SupConLoss) [25] to encourage the directions of subjects with the same label changes to be closer to one another. The label transitions considered are HC to HC, HC to MCI, and MCI to MCI. The process of calculating the group alignment loss is using equations5  6, where *N* represents the total number of subjects, and *L_G,i_* represents each group alignment loss for subject *i*. SupConLoss(⋅, ⋅) is the SupConLoss function. $l_{k-1,k}^{i}$ is the label transition type of subject *i* from (*k* − 1)th visit to *k*th visit.

(5) $$L_{G}=\sum_{i=1}^{N}L_{G,i}=\sum_{i=1}^{N}\sum_{k=2}^{V}\sum_{m_{k}=1}^{M_{i,k}}\sum_{m_{k-1}=1}^{M_{i,k-1}}SupConLoss\left(d_{i,k-1}^{m_{k-1},m_{k}},l_{k-1,k}^{i}\right)$$

(6) $$l_{k-1,k}^{i}=\left\{ \begin{array}{l} 0,\;label\;is\;HC\;both\;in\;{\left(k-1\right)}^{th}\;and\;k^{th}\;visit\;\;\;\;\;\;\;\;\;\;\;\;\;\;\;\;\;\;\;\;\;\;\;\;\;\;\;\;\;\;\;\;\\ 1,\;label\;is\;HC\;in\;{\left(k-1\right)}^{th}\;but\;later\;becomes\;MCI\;in\;k^{th}\;visit\\ 2,\;label\;is\;MCI\;both\;in\;{\left(k-1\right)}^{th}\;and\;k^{th}\;visit\;\;\;\;\;\;\;\;\;\;\;\;\;\;\;\;\;\;\;\;\;\;\;\;\;\;\;\;\;\; \end{array}\right.$$

To summarize, group alignment loss *L_G_* aims to minimize the distance between the cognitive progression directions of different subjects with the same cognitive label transitions types. This helps ensure that subjects exhibiting similar cognitive changes have consistent representations, thereby improving the model’s ability to generalize across different subjects. By leveraging SupConLoss, we can effectively capture and align these progression patterns, leading to more accurate and robust cognitive state predictions.

### Classifier and Overall Loss

After the ATM, we obtain the final outputs $\left\{{\overset{-}{e}}_{i,1}^{m_{1}},{\overset{-}{e}}_{i,2}^{m_{2}},{\overset{-}{e}}_{i,3}^{m_{3}},d_{i,1}^{m_{1},m_{2}},\;d_{i,2}^{m_{2},m_{3}}\right\}$, where ${\overset{-}{e}}_{i,k}^{m_{k}}$ represents the enhanced latent representation after the cross-attention layer. These representations are then concatenated and fed into a classifier. The classifier comprises a batch normalization layer and a fully connected layer to produce the final prediction. Finally, the overall loss function can be formulated by equation 7, where $L_{class}$ is the binary classification loss, and α, δ, and γ are the hyperparameters of loss weight for *L_E_*, *L*_*G*_, and *L_S_*, respectively. The hyperparameters are fine-tuned, and the optimal values are 0.01, 0.001, and 0.001.


(7) $$L=\;L_{class}+\alpha L_{E}+\delta L_{G}+\gamma L_{S}$$

The encoder loss *L_E_* is a contrastive loss designed to enhance the representativeness of the embeddings generated by the encoder and is defined by equation 8, where *N* is the total number of the subjects, *V* is the total number of visits, and *M_k_* is the total number of stories in the *k*th visit. This loss ensures that embeddings with the same cognitive status are closer to one another while pushing apart embeddings with different labels.


(8) $$L_{E}=\sum_{i=1}^{N}L_{E,i}=\sum_{i=1}^{N}\sum_{k=2}^{V}\sum_{m_{k}=1}^{M_{k}}SupConLoss\left(e_{i,k}^{m_{k}},l_{i,k}\right)$$

### Dataset

#### Dataset Collection

Our experiments primarily focus on comparisons using our self-collected National Taiwan University Autobiographical Memory Test (NTU-AM) dataset. Previous studies [6,11,26,27] have confirmed that the AM test effectively tracks changes in cognitive function by evaluating subjects’ memory functions. It can be used as a tool for MCI testing. During the test, subjects are asked to recall important life stories from different periods in their past. Compared with formal memory testing methods, this process resembles a general conversation, which helps reduce the stress experienced by subjects.

For the collected data used in this study, professional psychologists conducted one-on-one interviews. During these interviews, a microphone is used to record the conversation between the subject and the interviewer. Furthermore, subjects are asked first to talk about the titles of the important events they want to share (eg, “I want to share the story about when I was admitted to college at the age of 18 and my test result were announced”). The process of describing the events can be divided into two stages, namely, recall and probing.

1. Recall: The subject is first asked to roughly describe the events leisurely and then is encouraged to share as many details of the event as possible.
2. Probing: The interviewer then asks even more detailed questions based on what the subject mentioned in the recall stage. These questions cover various categories, such as “what,” “when,” “where,” emotion or thought, and time integration.

During a single interview, the same subject can share multiple stories as a matter of fact. To sum up, the overall process of the AM interview test can be described as follows: (1) the subject decides the number and content of stories to be shared, (2) shares the recall content of each story, and then (3) the interviewer probes each story with a series of detailed questions.

Moreover, to track the cognitive changes of subjects over a long term, they are invited to take the AM test again after at least 6 months. In our current research, our longitudinal data include up to 3 rounds of AM interview test data, that is, each subject has 1 test data and 2 follow-up data. All data were collected by professional psychologists with institutional review board approval, ensuring high-quality data.

The National Taiwan University Autobiographical Memory Test Cross-Sectional (NTU-AM-CS) dataset was collected under a cross-sectional scenario, with the demographic distribution of participants shown in Table 1. This dataset includes 111 subjects, consisting of 56 HC and 55 MCI participants. Each participant could provide multiple important life stories during a single visit.

**Table 1.** The demographic distribution of participants in the National Taiwan University Autobiographical Memory Test Cross-Sectional dataset.

Group	N	Male	Female	Age (years), mean (SD)	Years of education, mean (SD)	MMSE^a^, mean (SD)
HC^b^ (n=56)	56	18	38	71.3 (5.4)	16.2 (9.8)	28.7 (1.6)
MCI^c^ (n=55)	55	17	38	70.7 (6.9)	13.1 (7.1)	27.1 (2.0)
Total (N=111)	111	35	76	71.0 (6.2)	14.6 (8.7)	27.9 (2.0)

a MMSE: Mini-Mental State Examination.

b HC: healthy control.

c MCI: mild cognitive impairment.

The longitudinal scenario dataset, National Taiwan University Autobiography Memory Test Longitudinal (NTU-AM-LG), involves 54 of the original 111 subjects who participated in follow-up studies, including 28 HC and 26 MCI participants. The types of data collected are the same as in the cross-sectional scenario, encompassing both recall and probing audio files. The key difference is that NTU-AM-LG includes story audio files from 3 visits. The demographic distribution for this dataset is also shown in Table 2.

**Table 2.** The demographic information of the National Taiwan University Autobiography Memory Test Longitudinal dataset.

Group (N=54)	Male	Female	Age (years), mean (SD)	Years of education, mean (SD)	MMSE^a^, mean (SD)
HC^b^ (n=28)	12	16	74.0 (5.9)	14.3 (2.7)	28.4 (1.5)
MCI^c^ (n=26)	7	19	72.5 (6.4)	14.0 (9.6)	27.5 (1.9)
Total	19	35	73.3 (6.1)	14.1 (7.0)	28.0 (1.8)

a MMSE: Mini-Mental State Examination.

b HC: healthy control.

c MCI: mild cognitive impairment.

#### Ethical Considerations

The collection of experimental data from human participants was reviewed and approved by the National Taiwan University Hospital Research Ethics Committee B (protocol no. 202105013RINB, approved on June 18, 2021). All participants provided written informed consent prior to data collection. The original consent and institutional review board approval explicitly permitted the secondary analysis of deidentified recordings and transcripts for research purposes, without requiring additional consent. All data were fully deidentified before analysis, and no personal or identifiable information was retained in the research dataset. Participant privacy was protected by storing all files on secure, access-restricted servers, and only aggregated results are reported. Participants received NTD 160 (equivalent to US $5.09) per hour as compensation for their time and contribution. No identifiable participant images are included in this manuscript.

#### Experiment Dataset

The summary of the collected dataset can be provided. The total number of datasets is shown in Table 3, where the unit of measurement is the number of stories. Since each story includes both recall and probing phases, the quantities are identical for recall and probing data.

**Table 3.** Primary dataset statistics.

Dataset	Visit 1		Visit 2		Visit 3		Total	
	HC^a^	MCI^b^	HC	MCI	HC	MCI	HC	MCI
NTU-AM-CS^c^	134	122	N/A^d^	N/A	N/A	N/A	134	122
NTU-AM-LG^e^	44	30	50	40	76	54	170	124

a HC: healthy control.

b MCI: mild cognitive impairment.

c NTU-AM-CS: National Taiwan University Autobiography Memory Test Cross-Sectional.

d N/A: not applicable.

e NTU-AM-LG: National Taiwan University Autobiography Memory Test Longitudinal.

NTU-AM-CS is a cross-sectional dataset that includes a total of 111 subjects (56 HCs and 55 with MCI). Each subject participated in a single AM interview, during which they could share multiple stories. Therefore, NTU-AM-CS does not include repeated follow-up visits.

NTU-AM-LG is the longitudinal subset derived from NTU-AM-CS. A portion of participants agreed to return for additional follow-up interviews, resulting in up to 3 visits per subject. As shown in Table 3, the number of stories per visit varies because the volume of data depends on how many stories participants chose to share during each session. In some cases, more stories were collected in visit 3 than in earlier visits, which explains the higher data volume observed.

To further clarify disease progression patterns, the number of subjects transitioning across cognitive status categories between visits, including stable (HC→HC, MCI→MCI) and progressive or recovered (HC→MCI, MCI→HC) cases, is shown in Table 4.

**Table 4.** Cognitive status transitions between consecutive visits in the National Taiwan University Autobiography Memory Test Longitudinal dataset across visits 1 and 2 and visits 2 and 3.

Transition	Number of cases (subject based), n =108
HC^a^ to HC	50
HC to MCI^b^	10
MCI to HC	8
MCI to MCI	40

a HC: healthy control.

b MCI: mild cognitive impairment.

For additional experiments, we primarily used a semistructured speech dataset. The National Taiwan University Hospital Picture Description Test (NTUH-PIC) and National Taiwan University Hospital Memory Test (NTUH-MEM) datasets, which we collected ourselves, include speech data from picture description and logical memory tests, respectively. Both datasets feature 2 cognitive labels: HC and MCI. In the picture description task, participants are required to describe the scenario depicted in a specific picture as accurately and detailed as possible. In the NTUH-PIC dataset, the well-known “cookie theft” picture [28] is used. For the logical memory test, participants first listen to a story and then must repeat it from memory. The third dataset, ADReSSo [29], was introduced as part of the cognitive challenge at INTERSPEECH 2020. This dataset also involves a picture description test, which also used “cookie theft” [28] but differs from the other datasets in that it includes only 2 cognitive labels: HC and AD. Due to data access limitations, we used only the training set for evaluation in this case. By incorporating these additional datasets, we aim to comprehensively evaluate the robustness and generalizability of our model across various types of speech data and cognitive assessments. The statistics of the additional dataset are shown in Table 5.

**Table 5.** Additional dataset statistics.

Dataset	Total	HC^a^	MCI^b^	Language	Test
NTUH-PIC^c^	80	40	40	Chinese	Picture Description Test
NTUH-MEM^d^	104	52	52	Chinese	Logical Memory Test
ADReSSo (Train)	166	79	87	English	Picture Description Test

a HC: healthy control.

b MCI: mild cognitive impairment.

c NTUH-PIC: National Taiwan University Hospital Picture Description Test.

d NTUH-MEM: National Taiwan University Hospital Memory Test.

### Experiments

We conducted a series of experiments to evaluate our proposed longitudinal multimodal model under different data conditions and to verify the contribution of each model component.

#### Experiments on NTU-AM-LG

We evaluated the model on the longitudinal NTU-AM-LG dataset using 2 speaking tasks: recall and probing. For each task, we compared four variants of our model:

1. Ours (audio): acoustic branch only, wav2vec 2.0 to acoustic encoder to classifier.
2. Ours (text): linguistic branch only, BERT to linguistic encoder to classifier.
3. Ours (CS): cross-sectional problem setting
4. Ours (LG): full proposed longitudinal multimodal model with direction encoding and alignment losses.

These models were compared against standard baselines (BERT, wav2vec 2.0, and the prior AM-based work [11]), and the corresponding results are reported in the “Results” section.

An ablation study on NTU-AM-LG is made to examine the contribution of the ATM, direction embedding, and the 2 alignment losses, and we performed ablation experiments by removing (1) the direction, (2) the subject alignment loss, (3) the group alignment loss, and (4) both alignment losses.

#### Experiments on NTU-AM-CS

To demonstrate that the proposed encoders and fusion strategy also work without longitudinal information, we trained the same acoustic and linguistic encoders in the cross-sectional problem setting (Our [CS]) and compared them with BERT, wav2vec 2.0, and the prior AM method [11]. An ablation study is also conducted to highlight the importance of linguistic features and context embeddings.

#### Experiments on Additional Datasets

We further tested whether our proposed model transfers to other cognitive-speech datasets (NTUH-PIC and NTUH-MEM). We compared our method with that of Lin et al [18].

Finally, to enable comparison with existing AD or MCI speech work on a public dataset, we evaluated our model on the ADReSSo corpus.

#### Experimental Setup

In the subsequent experiments, due to the limitations of the dataset size, we will present the averaged performance of 10-fold cross-validation. To avoid data leakage, we will split the training set and validation set by subject for the NTU-AM-CS and NTU-AM-LG datasets. It ensures that audio files from different visits of the same subject will not appear in the training set and validation set simultaneously.

For the NTUH-PIC, NTUH-MEM, and ADReSSo datasets, since each subject provides only 1 audio file, there is no need to split the data by subject. We will perform down-sampling on the majority class in the training set during the experimental process to stabilize the model’s learning capability. All experiments are conducted on an NVIDIA RTX 4090 GPU.

#### Evaluation Metrics

In the experimental section, we will use 6 different evaluation metrics to measure the performance of the model: accuracy, *F*_1_-score, precision, sensitivity (recall), specificity, and AUROC. These are common indicators used to assess the predictive ability of a model. Each metric provides a different perspective on the effectiveness of the model.

In the NTU-AM dataset, since the dataset is split by subject, each evaluation set from every fold may have a similar number of subjects, but the number of audio files provided by each subject can vary. This can result in label imbalance in the final soft-voting results. Therefore, the most critical metrics in the subsequent experiments will be *F*_1_-score and AUROC. These 2 metrics provide a fairer assessment in situations with label imbalance. As for other experiments, the primary metrics of interest will be accuracy and *F*_1_-score.

#### Baseline

In the subsequent experiments in the NTU-AM dataset, we will use a fine-tuned BERT model and the wav2vec 2.0 model as baselines. Since these 2 models are the backbones of our proposed model, in the NTU-AM-LG dataset, the baseline model will be composed of these 2 main models as encoders. The embeddings generated from the 3 visits will be concatenated and passed through the same classifier. For the NTU-AM-CS dataset, the backbone model will serve as the encoder, followed by a classifier. However, these models will not have additional loss functions to assist training during the training process, relying only on the basic binary cross-entropy loss.

#### Implementation Details

The probing data are interview data, which include both the interviewer’s and the subject’s voices. Since our study aims to determine whether the subject has a tendency for MCI based on their audio data, the interviewer’s speech is considered noise during training. To remove the interviewer’s portion, we use the tool kit “pyannote” to split the audio into different speaker segments. This process results in audio files without the interviewer’s voice. These cleaned files are then used to generate the linguistic features during the data preprocessing stage.

We used the “openai/whisper-large-v2” version from Hugging Face for the ASR tool. For data preprocessing, the pretrained Wav2vec 2.0 model used for the English dataset is “facebook/wav2vec2-base-960h,” while for the Chinese dataset, we used “jonatasgrosman/ wav2vec2-large-xlsr-53-chinese-zh-cn,” which are also from HuggingFace. We used a 15-second clip segmentation with a 5-second overlap. Considering the limitations of the Wav2vec model, we resampled the audio files to 16,000 Hz and standardized them to mono-channel.

For the linguistic model, the BERT model used for the English dataset is “google-bert/bert-base-uncased,” and for the Chinese dataset, it is “hfl/chinese-bert-wwm-ext,” which is also the version used for the BERT baseline. In the Wav2vec baseline model, we used “jonatasgrosman/wav2vec2-large-xlsr-53-chinese-zh-cn.” The hyperparameters used for implementing the proposed model are shown in Table 6.

**Table 6.** Hyperparameters used for implementation.

Hyperparameters	Value
BERT^a^ attention dropout rate	0.3
BERT linear layer dropout rate	0.3
Maximum sentence (token) length	512
Dimension of audio embedding	128
Dimension of text embedding	128
Dimension of direction embedding	32
Learning rate	1e-5
Number of layers for attention-fusion	1
Number of layers for cross-attention	1
Batch size for training	8
Batch size for evaluation	8
Maximum epoch number	150
Early stop	10
α for encoder loss	0.01
δ for group alignment loss	0.001
γ for subject alignment loss	0.001

a BERT: bidirectional encoder representation for transformer.

## Results

### Results of NTU-AM-LG

We experiment with the model’s effectiveness on both recall and probing data. Given the limited research on longitudinal speech, we primarily compare our model against baselines such as BERT, Wav2vec 2.0, and the unimodal model within our longitudinal framework. In addition, we present an ablation study to demonstrate the necessity of each component in our model. We discuss the results for recall data and probing data separately. It is important to note that both the cross-sectional (CS) and longitudinal (LG) models are trained on the same NTU-AM-LG dataset, sharing identical subjects and feature representations. The difference lies only in the problem formulation: the CS model performs single-visit classification, while the LG model performs a prognostic task that leverages prior visit embeddings to predict the final cognitive state. Thus, the performance difference reflects the use of incorporating longitudinal context rather than architectural or data disparities.

Table 7 shows the performance of our proposed model and the baselines for the recall data. First, when the input consists only of audio data, our proposed model “Ours (audio)” outperforms the previous work [11] on most metrics. Notably, there are improvements of 5% and 6% in *F*_1_-score and AUROC, respectively.

**Table 7.** Results of recall data on National Taiwan University Autobiography Memory Test Longitudinal dataset.

Method	Accuracy	*F*_1_-score	Precision	Sensitivity	Specificity	AUROC^a^
Audio only						
Chang et al [11]	0.66	0.64	0.74	0.67	0.65	0.63
Wav2Vec2	0.59	0.58	0.53	0.78^b^	0.43	0.60
Ours (audio)	0.67	0.69	0.73	0.73	0.66	0.69
Text only						
BERT^c^	0.73	0.65	0.77	0.64	0.82	0.73
Ours (text)	0.75	0.72	0.83	0.73	0.72	0.73
Text + Audio						
Ours (CS^d^)	0.72	0.72	0.68^b^	0.76^b^	0.74	0.66
Ours (LG^e^)	0.82^b^	0.78^b^	0.95^b^	0.72^b^	0.97^b^	0.85^b^

a AUROC: area under the receiver operating characteristic curve.

b Highest value for each metric.

c BERT: bidirectional encoder representation for transformer.

d CS: cross-sectional.

e LG: longitudinal.

As for the model using only linguistic data as input, our proposed linguistic longitudinal model, denoted as “Ours (text),” outperforms BERT, demonstrating that including extra-linguistic features and context embedding helps improve the model’s performance with 7% increase in the *F*_1_-score.

Finally, our model, which is denoted as “Ours (LG)” in Table 7, significantly outperforms other models across most metrics for the dual-modal longitudinal model that combines both data types. Specifically, there is up to a 16% improvement in accuracy, a 14% improvement in *F*_1_-score, and a 22% improvement in AUROC. Since the previous work used only unimodal data, examining AM data from different perspectives benefits MCI detection. We also compare the cross-sectional setting results of NTU-AM-LG, denoted as “Ours (CS)” in Table 7, with the longitudinal settings, confirming that long-term tracking of subjects’ cognitive trajectories in NTU-AM-LG helps improve the model’s prediction accuracy. Specifically, there are improvements of 10% in accuracy, 6% in *F*_1_-score, and 19% in AUROC.

Table 8 shows the results of the probing data. For the audio data, our proposed model outperforms wav2vec 2.0 in most metrics, with improvements of 9%, 18%, and 10% in accuracy, *F*_1_-score, and AUROC, respectively. This indicates that instead of directly using wav2vec 2.0’s output representation for classification, extracting features using wav2vec 2.0 followed by a convolutional neural network–based acoustic encoder structure can better capture the hidden cognitive patterns in audio data.

**Table 8.** Result of probing data on National Taiwan University Autobiography Memory Test Longitudinal dataset.

Method	Accuracy	*F*_1_-score	Precision	Sensitivity	Specificity	AUROC^a^
Audio only						
Wav2vec2	0.76	0.53	0.60	0.53	0.87	0.70
Ours (audio)	0.85	0.71	0.67	0.78	0.83	0.80
Text only						
BERT^b^	0.85	0.72	0.83	0.71	0.94	0.82
Ours (text)	0.84	0.80	0.86	0.81	0.82	0.82
Text + Audio						
Ours (CS^c^)	0.71	0.67	0.61	0.75	0.63	0.68
Ours (LG^d^**)**	0.89^e^	0.83^e^	0.90^e^	0.82^e^	0.95^e^	0.89^e^

a AUROC: area under the receiver operating characteristic curve.

b BERT: bidirectional encoder representation for transformer.

c CS: cross-sectional.

d LG: longitudinal.

e Values in boldface are the highest values for each metric.

For the linguistic data results, our model performs comparably with BERT, with improvements of 8%, 3%, and 10% in *F*_1_-score, precision, and sensitivity, respectively. This suggests that the inclusion of linguistic features and the context embedding layer can also aid in MCI detection on probing data.

Finally, in the dual-modal data tests, the proposed longitudinal model achieved the best performance, excelling in every metric. Specifically, it showed improvements of up to 13%, 30%, and 19% in accuracy, *F*_1_-score, and AUROC, respectively. Comparing the longitudinal model with the cross-sectional model, it is evident that the longitudinal model continues to perform better. This indicates that incorporating input from multiple time points and considering the concept of aging direction can enhance the model’s predictions, resulting in performance increases of 12%, 16%, and 21% in accuracy, *F*_1_-score, and AUROC, respectively.

#### Ablation Study

In the longitudinal setting, the most crucial idea is the alignment loss we proposed. Therefore, this section examines the contribution of alignment loss by removing each loss individually. The results are shown in Table 9. In the table, “Without direction” refers to not including direction embedding as the classifier input; namely, the input would be $e_{i,1}^{m_{1}}$, $e_{i,2}^{m_{2}}$, $e_{i,3}^{m_{3}}$ treating each time point’s data as independent.

**Table 9.** Result of the ablation study for our proposed model on National Taiwan University Autobiography Memory Test Longitudinal dataset.

Method	Accuracy	*F*_1_-score	Precision	Sensitivity	Specificity	AUROC^a^
Recall data						
Without direction	0.79	0.74	0.71	0.83	0.67	0.75
Ours (LG^b^**)**	0.82^c^	0.78^c^	0.95^c^	0.72	0.97^c^	0.85^c^
Subject alignment loss	0.71	0.67	0.68	0.77	0.63	0.70
Group alignment loss	0.68	0.69	0.68	0.89^c^	0.47	0.68
Both alignment loss	0.65	0.59	0.64	0.62	0.63	0.63
Probing data						
Without direction	0.79	0.70	0.68	0.76	0.78	0.77
Ours (LG)	0.89^c^	0.83^c^	0.9^c^	0.82^c^	0.95^c^	0.89^c^
Subject alignment loss	0.78	0.59	0.61	0.59	0.92	0.75
Group alignment loss	0.69	0.54	0.63	0.55	0.8	0.68
Both alignment loss	0.65	0.53	0.60	0.53	0.87	0.70

a AUROC: area under the receiver operating characteristic curve.

b LG: longitudinal.

c Highest value for each metric in recall data and probing data, respectively.

The results show that incorporating alignment losses with direction embedding improves performance across both recall and probing data. For instance, on recall data, our full model improves AUROC by 10% (0.85 vs 0.75) and *F*_1_-score by 4% compared with “Without direction.” On probing data, the gains are even larger, with AUROC improved by 12% (0.89 vs 0.77) and *F*_1_-score improved by 13% (0.83 vs 0.70). Importantly, removing the group alignment loss results in the sharpest performance drop, with AUROC decreasing by 17% (0.68 vs 0.85) and 21% (0.68 vs 0.89) for recall and probing data, respectively. Similarly, removing both alignment losses further reduces AUROC to 0.63 (recall) and 0.70 (probing), confirming their necessity.

Overall, these results highlight the complementary importance of both alignment losses that rely on the strong supervisory signal of ground-truth label transitions to capture temporal progression patterns in longitudinal settings. Removing either the subject alignment loss or the group alignment loss results in significant performance degradation, and removing both leads to the sharpest decline. This indicates that while the subject alignment loss stabilizes intrasubject trajectory consistency, the group alignment loss aligns intersubject cognitive progression trends across similar diagnostic groups.

#### Visualization

**Figure 9.**
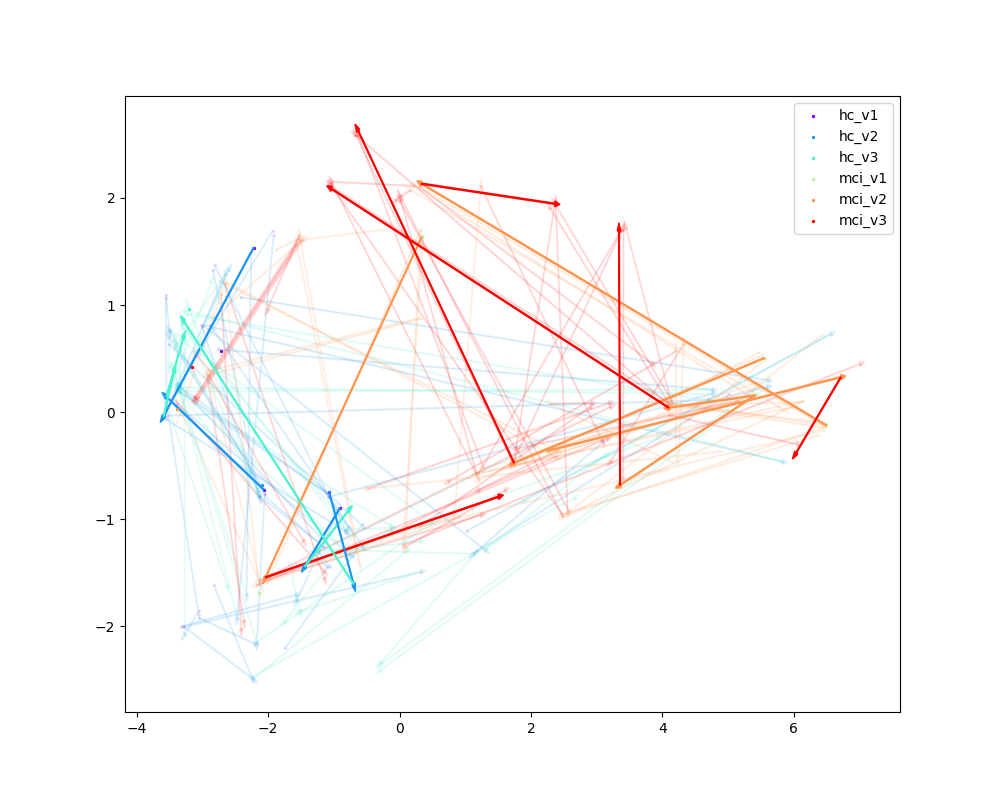
Principal component analysis projection of subject embeddings without alignment loss. The x- and y-axes represent the first 2 principal components. Colors denote diagnostic group and visit (blue tones = healthy control visits 1‐3; red tones = mild cognitive impairment visits 1‐3). Arrows illustrate the trajectory of each subject’s embeddings. The scattered trajectories reflect high variability and weak temporal consistency without alignment loss.

We visualize the latent space distribution of subjects across different visits to better interpret the role of the alignment loss. After passing through all modules, we extracted the final embeddings, applied principal component analysis for dimensionality reduction, and projected the results into a 2D space. The arrows in Figures9  10 represent the trajectory of each subject’s embeddings across consecutive visits, thereby illustrating how cognitive states evolve over time.

**Figure 10.**
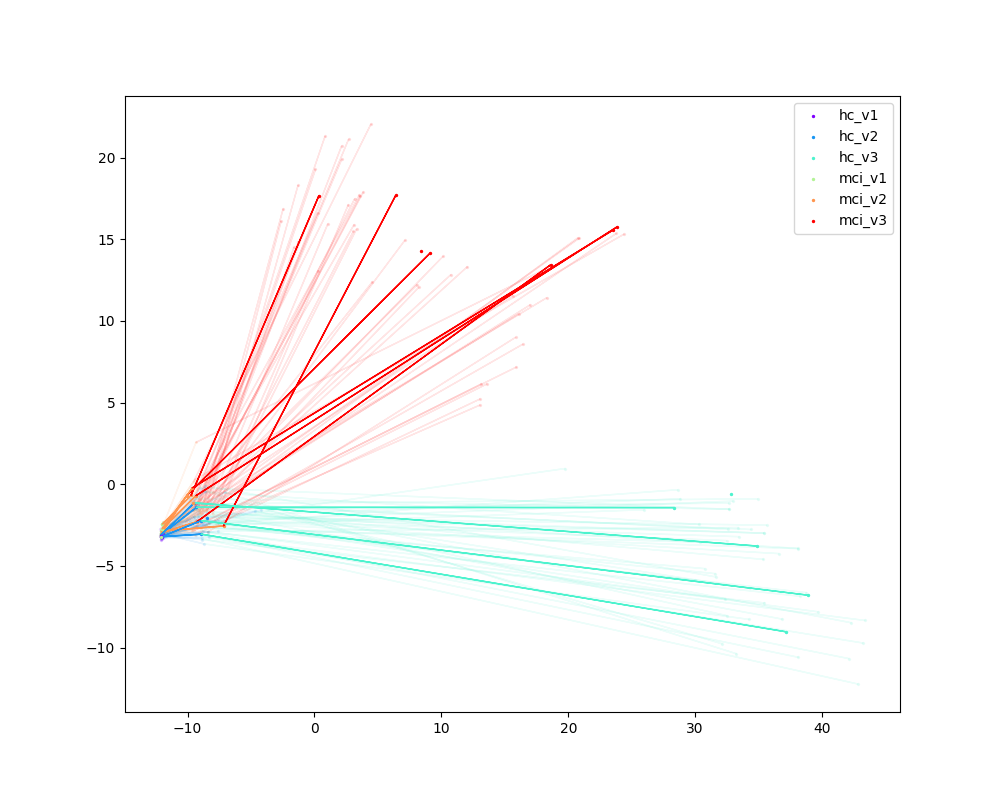
Principal component analysis projection of subject embeddings with alignment loss. The x- and y-axes show the first 2 principal components. Colors indicate diagnostic group and visit (blue tones = healthy control visits 1‐3; red tones = mild cognitive impairment visits 1‐3). Arrows illustrate the trajectory of each subject’s embeddings. With alignment loss, trajectories become far more coherent and directionally consistent, indicating improved temporal stability and clearer separation between healthy control and mild cognitive impairment progression patterns.

As shown in Figure 9 (without alignment loss), the trajectories are scattered and inconsistent, with large variability both within and across groups. This makes it difficult to distinguish between HC and MCI or to capture stable temporal patterns. In contrast, Figure 10 (with alignment loss) reveals more coherent trajectories: subjects within the same group (HC or MCI) show consistent progression directions across visits, while the separation between HC and MCI becomes clearer. Importantly, this indicates that the alignment loss encourages the model to capture cognitive progression trends rather than random variability in story content, reflecting interpretable patterns of decline (for MCI) and stability (for HC).

These results demonstrate that the alignment loss not only improves metrics but also enhances interpretability by aligning temporal trajectories in a way that reflects clinically meaningful differences in cognitive change. Feature-level analyses could provide even more intuitive examples of how the ATM aligns specific cognitive markers in speech. Future feature-level analyses could further enhance this by providing more intuitive illustrations of how the ATM captures and aligns specific cognitive markers in speech.

### Results of NTU-AM-CS

In addition to the main longitudinal experiment, we conducted further evaluations in a cross-sectional setting to assess the performance of our proposed model. Here, the primary comparison is with our previous work [11], which also used audio and text data. However, a key difference is that the prior work relied on manual transcripts, which are less scalable and require special preprocessing for the transcripts. The results are shown in Tables10  11.

**Table 10.** Results of recall data on National Taiwan University Autobiographical Memory Test Cross-Sectional dataset^c^.

Method	Accuracy	*F*_1_-score	Precision	Sensitivity	Specificity	AUROC^a^
Audio only						
Wav2vec2	0.65	0.42	0.41	0.48	0.47	0.51
Ours (audio)	0.57	0.60	0.52	0.72	0.45	0.58
Text only						
BERT^b^	0.67	0.56	0.62	0.61	0.67	0.64
Ours (text)	0.69	0.66	0.65	0.68	0.69	0.69^c^
Text + Audio						
Chang et al [11]	0.75^c^	0.61	0.76^c^	0.55	0.85^c^	0.68
Ours (CS^d^)	0.74	0.72^c^	0.74	0.69^c^	0.83	0.68

a AUROC: area under the receiver operating characteristic curve.

b BERT: bidirectional encoder representation for transformer.

c Highest value of each evaluation metric.

d CS: cross-sectional.

**Table 11.** Results of probing data on National Taiwan University Autobiographical Memory Test Cross-Sectional dataset^c^.

Method	Accuracy	*F*_1_-score	Precision	Sensitivity	Specificity	AUROC^a^
Audio only						
Wav2vec2	0.55	0.49	0.65	0.43	0.66	0.61
Ours (audio)	0.61	0.65	0.56	0.84	0.43	0.64
Text only						
BERT^b^	0.69	0.64	0.66	0.72	0.67	0.69
Ours (text)	0.69	0.66	0.67	0.64	0.75**^c^**	0.65
Text + Audio						
Chang et al [11]	0.77**^c^**	0.64	0.62	0.73	0.68**^c^**	0.65
Ours (CS^d^)	0.73	0.71**^c^**	0.67**^c^**	0.77**^c^**	0.68	0.72**^c^**

a AUROC: area under the receiver operating characteristic curve.

b BERT: bidirectional encoder representation for transformer.

c Highest value of each evaluation metric.

d CS: cross-sectional.

Our proposed method performs better or comparably on critical metrics such as *F*_1_-score and AUROC in the results of recall data. Specifically, there is an 11% improvement in *F*_1_-score. However, the precision is not as good as in the study by Chang et al [11]; our sensitivity metric performs better. For probing data, our method also yields better results in *F*_1_-score and AUROC. However, the overall performance of the NTU-AM-CS dataset is not as promising as that of the NTU-AM-LG dataset. Detecting MCI features in AM data is challenging and reinforces the idea that long-term data relationships aid in model prediction.

#### Ablation Study

We investigated the contributions of linguistic features and context embedding in the encoder module for the prediction ability. The results are shown in Table 12. It demonstrates the impact of removing individual components on the model’s predictions. For example, when linguistic features are removed, there is a noticeable decrease in specificity and a 6% drop in *F*_1_-score. The most significant impact is seen when context embedding is removed, resulting in a 9% decrease in *F*_1_-score and substantial declines in most other metrics. Only the sensitivity metric performs better, but overall, the original configuration still performs best in terms of *F*_1_-score, which balances sensitivity and precision.

**Table 12.** Results of the ablation study for recall data on the National Taiwan University Autobiographical Memory Test Cross-Sectional dataset.

Method	Accuracy	*F*_1_-score	Precision	Sensitivity	Specificity	AUROC^a^
Ours (CS^b^)	0.74^c^	0.72^c^	0.74^c^	0.69^c^	0.83^c^	0.68^c^
Linguistic features	0.62	0.66	0.57	0.84^c^	0.45	0.62
Context embedding	0.59	0.63	0.53	0.80	0.45	0.60

a AUROC: area under the receiver operating characteristic curve.

b CS: cross-sectional.

c Highest values of each evaluation metric.

These results indicated that linguistic features provide auxiliary information that aids the model’s decision-making, particularly in identifying negative samples. Context embedding supports the model’s understanding of the story by distinguishing between the story topic and content through embeddings.

### Result of Additional Dataset

In the experiment with an additional dataset, our main goal was to confirm whether the method our model uses to encode data is also applicable to other datasets related to cognitive tests. This means whether the way we encoded each data contributes to MCI detection. In Table 13, we present several results related to the detection of MCI. Our primary comparison targets in the NTUH-PIC and NTUH-MEM datasets are as per the study by Lin et al [18], which use textual information, including pauses, for MCI prediction. From the NTUH-PIC experimental results, there was an improvement of 11% in accuracy and 18% in *F*_1_-score.

**Table 13.** Results of the National Taiwan University Hospital dataset.

Dataset	Method	Accuracy	*F*_1_-score	Precision	Sensitivity	Specificity	AUROC^a^
NTUH-PIC^b^	Lin et al [18]	0.68	0.63	0.81^c^	0.58	0.78^c^	N/A^e^
	Ours (CS^d^)	0.79^c^	0.81	0.80	0.88^c^	0.7	0.79
NTUH-MEM^f^	Lin et al [18]	0.68	0.66	0.75	0.7	0.65	N/A
	Ours (CS)	0.78^c^	0.76^c^	0.82^c^	0.73^c^	0.83^c^	0.78

a AUROC: area under the receiver operating characteristic curve.

b NTUH-PIC: National Taiwan University Hospital Picture Description Test.

c Highest values for each evaluation metric in its respective dataset.

d CS: cross-sectional.

e N/A: not applicable.

f NTUH-MEM: National Taiwan University Hospital Memory Test.

Similarly, in the NTUH-MEM dataset, a 10% improvement was observed in both accuracy and *F*_1_-score compared with the study by Lin et al [18], indicating overall better performance. This experiment demonstrates that our data-encoding method effectively extracts latent representations suitable for MCI detection. Viewing data from multiple modalities provides consistent benefits for MCI detection. In addition, the performance differences observed in the NTU-AM-CS experiment indicate that using AM data for MCI detection is relatively challenging.

Due to the nature of the self-collected dataset, making comparisons with other works is challenging. Therefore, we present results on an open dataset in the final experiment. However, the ADReSSo dataset provides only HC and AD data. Table 14 shows our results alongside those of other state-of-the-art methods. Overall, our approach yields the best results, with a 4% improvement in both accuracy and *F*_1_-score. In addition, our precision achieves the best performance. Once again, it confirms that our data-encoding method contributes to predictive outcomes in AD and MCI detection.

**Table 14.** Result of the ADReSSo dataset^a^.

Method	Accuracy	*F*_1_-score	Precision	Sensitivity	Specificity
Syed et al [30]	0.84	0.84	N/A^b^	0.79	0.90
Rohanian et al [31]	0.84	N/A	N/A	N/A	N/A
Zhu et al [32]	0.83	0.83	0.83	0.84^a^	N/A
Ying et al [33]	0.84	0.84	0.85	0.84^a^	N/A
Ours (CS^c^)	0.88^a^	0.88^a^	0.93^a^	0.84^a^	0.92^a^

a Highest value in each evaluation metric.

b N/A: not applicable.

c CS: cross-sectional.

## Discussion

### Principal Results

In this study, we proposed a longitudinal framework for MCI detection using AM test speech data. The key innovation is the ATM, which leverages direction embeddings with subject and group alignment losses to model cognitive changes across multiple visits.

Our experiments demonstrate that the longitudinal framework substantially outperforms cross-sectional approaches. Compared with single-visit models, the longitudinal model achieved notable improvements, with AUROC gains of up to 21%, *F*_1_-score increases of 16%, and accuracy improvements of 12%. These results confirm that incorporating multi–time point information provides a clearer picture of subtle cognitive trajectories that cannot be fully captured using only cross-sectional data.

The ablation studies further highlight the effectiveness of the alignment mechanisms. Removing the alignment losses led to considerable performance degradation, particularly a 17%‐21% drop in AUROC when the group alignment loss was excluded. This underscores the critical role of alignment in ensuring temporal consistency and improving the robustness of the framework.

In addition to evaluations on our self-collected dataset, we validated the framework on the publicly available ADReSSo dataset, which uses English picture description speech. Despite differences in language and task, our model achieved more than 88% accuracy, suggesting that the approach generalizes well across languages and cognitive assessment protocols.

In our analysis, the distribution of false positives and false negatives provides additional context for interpreting the model’s sensitivity and specificity. False positives, which reduce specificity, were often linked to healthy subjects who displayed atypical speech characteristics such as disfluencies, pauses, or reduced lexical richness that mimicked early cognitive decline. On the other hand, false negatives, which lower sensitivity, were frequently observed in MCI subjects who retained strong narrative skills or enriched their responses with contextual details, thereby masking subtle impairments. These observations illustrate how individual variability in speech patterns can differentially impact sensitivity and specificity, underscoring both the strengths and the boundary conditions of the framework. While the alignment-based approach improves overall robustness, further refinement of feature representations and the inclusion of personalized modeling strategies may help balance sensitivity and specificity more effectively.

Taken together, these results highlight both the methodological and clinical significance of the proposed approach. Improvements in AUROC of 10%‐15% can translate into earlier identification of at-risk individuals and a reduction in false negatives in real-world screening. This demonstrates the potential of longitudinal, dual-modal speech analysis as a noninvasive, scalable tool for long-term cognitive monitoring**,** with the flexibility to be integrated into routine memory clinic assessments or used as a low-cost prescreening solution to complement standardized neuropsychological testing.

### Limitations

Although the proposed framework shows promising performance for early MCI detection, several limitations remain. One major limitation of this study lies in the relatively small sample size of the NTU-AM-LG dataset, which includes only 54 subjects. Although each subject contributes multiple audio recordings across different visits, the limited number of unique participants poses inherent challenges for deep learning models, including a higher risk of overfitting, model instability, and low statistical power. As a result, while the proposed model demonstrates promising performance trends, its robustness and generalizability should be interpreted with caution. To mitigate these risks, we adopted strict subject-independent data splits and cross-validation to avoid data leakage and improve reliability. Nevertheless, future studies with larger and more diverse longitudinal datasets are essential to confirm the model’s generalization capability and to further validate its clinical applicability. Furthermore, we also validate our approach on the publicly available ADReSSo dataset, although it is limited to a cross-sectional study. Cross-lingual robustness also presents a challenge, as our dataset is in Chinese while ADReSSo is in English. Linguistic and cultural factors such as narrative structure, emotional expression, and lexical choices may influence AM speech patterns and, in turn, affect model generalizability. Nevertheless, our results demonstrate encouraging cross-lingual generalizability. We highlight this as an open challenge and a direction for future work, where cross-lingual adaptation and multilingual modeling will be crucial to improving robustness across diverse populations.

Moreover, the current longitudinal dataset includes 3 visits per participant, which restricts the ability to capture long-term trajectories of cognitive change. Collecting data across additional time points would allow the development of models better suited to characterizing the progression of cognitive decline.

This study also acknowledges potential confounding factors arising from the use of automatic speech processing tools. The *whisper-timestamped* model and *pyannote* diarization tool kit were used for transcription and speaker segmentation, respectively; however, the resulting ASR and diarization errors were not formally measured. Such errors, especially the higher ASR error rates likely to occur in participants with MCI due to disfluency or reduced articulation, and diarization inaccuracies that may introduce noise, could impact the extracted linguistic and acoustic features. These unaddressed confounders represent an inherent limitation of this study.

### Further Studies

Based on the current findings, several directions can be explored in future work. First, extending the framework to multilingual settings will be essential for improving cross-lingual generalizability, as language and cultural factors strongly influence AM narratives. Second, adapting the model for low-resource scenarios will enhance its applicability in real-world clinical practice, where large-scale longitudinal datasets are often unavailable. Techniques such as transfer learning, data augmentation, and self-supervised pretraining could make the system more robust under such constraints.

Third, integrating AM speech analysis with other noninvasive modalities, such as wearable sensor data, offers the potential to capture complementary behavioral and physiological signals (eg, activity levels, sleep patterns, and heart rate variability). Such multimodal fusion could provide a more holistic view of cognitive health and improve early detection of decline. Finally, future studies should focus on personalized modeling of cognitive trajectories, acknowledging that the pace and pattern of decline vary across individuals. Personalized approaches may increase the clinical relevance of the framework and support tailored interventions.

### Conclusions

We developed a dual-modal longitudinal analysis system to enhance the accuracy of diagnosing MCI. The system consists of an encoder and an ATM. We used a pretrained Wav2vec 2.0 model for speech data and BERT for text data to generate latent representations. Our proposed ATM generates significantly improved performance for MCI prediction, with *F*_1_-scores of 0.78 and 0.83 on the recall and probing datasets, respectively. Combining data from both modalities improved understanding of the AM test data, with up to 19% and 12% improvements in *F*_1_-score compared with unimodal models. In addition, ablation experiments verified the necessity of context embedding and linguistic features in the linguistic encoder. Our model’s effectiveness was consistently superior across different datasets.

## Abbreviations

AD: Alzheimer disease
AM: autobiographical memory
ASR: automatic speech recognition
ATM: aging trajectory module
AUROC: area under the receiver operating characteristic curve
BERT: bidirectional encoder representation for transformer
CS: cross-sectional
LG: longitudinal
MCI: mild cognitive impairment
NTU-AM: National Taiwan University Autobiographical Memory Test
NTU-AM-CS: National Taiwan University Autobiographical Memory Test Cross-Sectional
NTUH-MEM: National Taiwan University Hospital Memory Test
NTUH-PIC: National Taiwan University Hospital Picture Description Test
POS: part-of-speech
